# Supraspinatus tendon transosseous vs anchor repair surgery: a comparative study of mechanical recovery in the rabbit

**DOI:** 10.1186/s13018-020-02085-8

**Published:** 2020-12-07

**Authors:** Joaquim Chaler, Hakim Louati, Hans K. Uhthoff, Guy Trudel

**Affiliations:** 1 Department of Physical Medicine and Rehabilitation, Egarsat, Terrassa, Barcelona, Spain; 2 Escola Universitaria de Salut i Esport, Universitat de Girona-Universitat de Barcelona, Campus Bellvitge, Hospitalet, Barcelona, Spain; 3 Bone and Joint Research Laboratory, The Ottawa Hospital Research Institute, 451 Smyth Road, Ottawa, Ontario K1H 5M2 Canada; 4 Division of Orthopedic Surgery, Department of Surgery, University of Ottawa, Ottawa, Ontario Canada; 5 Division of Physical Medicine and Rehabilitation, Department of Medicine, Department of Biochemistry, Microbiology and Immunology, University of Ottawa, Ottawa, Canada

**Keywords:** Supraspinatus tendon, Mechanical testing, Tendon injuries, Animal model, Rotator cuff, Rehabilitation

## Abstract

**Background:**

Supraspinatus (SSP) tendon ruptures requiring surgical repair are common. Arthroscopic suture anchor fixation has gradually replaced transosseous repair in supraspinatus tendon tear. Our objective was to compare mechanical properties between transosseous and anchor supraspinatus repair in the first 6 postoperative weeks in a rabbit model.

**Methods:**

One hundred and fifty-two rabbits had one supraspinatus tendon repaired either with an anchor suture 1 week after detachment or with transosseous sutures. Rabbits were euthanized at 0, 1, 2, 4 or 6 postoperative weeks. Experimental and contralateral tendons (304 tendons) were mechanically tested to failure. Data are expressed as percent of contralateral.

**Results:**

Anchor repair had higher loads to failure compared to transosseous repair, at immediate repair (week 0, 52 ± 21% vs 25 ± 17%, respectively; *p* = 0.004) and at 1 postoperative week (64 ± 32% vs 28 ± 10%; *p* = 0.003) with no difference after 2 weeks. There was no difference in stiffness. Transosseous repairs showed higher rates of midsubstance failures compared to anchor repairs at 1 (*p* = 0.004) and 2 postoperative weeks (*p* < 0.001). Both transosseous and anchor repairs restored supraspinatus mechanical properties after 4 postoperative weeks.

**Conclusion:**

Anchor repair provided better initial tensile strength while transosseous repair led to a faster normalization (namely, midsubstance) of the mode of failure. Research to optimize supraspinatus repair may need to consider the advantages from both surgical approaches.

## Background

Rotator cuff repair is one of the most commonly performed upper limb surgery [1, 2]. The past decades have seen a major shift in surgical technique not only from open to arthroscopic but also from transosseous to anchor repair [3]. Clinical studies and meta-analyses have found no superiority of one repair technique over the other with regards to functional outcome, pain scores, re-tear rate, or incidence of adhesive capsulitis [4–7]. But patient-based outcomes of pain, strength, range of motion, stability, or medical imaging indirectly assess tendon strength. Data on the restoration of mechanical strength after tendon to bone attachment with each surgical technique are necessary to produce evidence-based recommendations. Directly assessing tendon biomechanical properties is possible in experimental studies [8].

Similar to clinical data, no experimental evidence supports either the transosseous or the anchor superior mechanical strength during healing. Cadaveric studies were conducted but they solely inform on the mechanical properties of the initial construct without evidence on the postoperative period. In seven cadaveric studies (human and animal), four were unable to demonstrate differences between transosseous and anchor repairs [9–12]. Two reported significantly higher loads in transosseous-equivalent repairs [13, 14] and one found anchor repairs to be stronger [15]. These cadaveric studies fail to study the postoperative new enthesis formation with progressive mechanical restoration which is critically important to ensure long-term surgical success [16].

With no basic evidence to support superiority of the mechanical strength of transosseous or suture anchors after repair, direct experimental comparison of data on strength, stiffness, and mode of failure during the early postoperative phases was needed. In previous experiments, we detached one supraspinatus (SSP) tendon in rabbits [16, 17]. In one cohort, we reattached the SSP with transosseous sutures and in the other cohort with suture anchors and mechanically tested the rabbits at 0, 1, 2, 4 or 6 weeks. Our objective was to compare the biomechanical data from both studies. Since suture anchor repair is the current standard of care, we tested the following hypotheses: (1) SSP anchor repairs have better initial suture load at failure than transosseous repairs and (2) SSP anchor repairs reach normal load at failure faster than transosseous repairs. The results could help refine the rehabilitation program after SSP tendon repair.

The clinical relevance of this investigation testing the mechanical properties of supraspinatus tendon transosseous vs anchor repair surgery fits into the framework of translational orthopedic: how to fill the gap between basic sciences and clinical sciences [18–20].

## Methods

We compared data from two previous published investigations [16, 17]. Direct comparative analysis of data is essential to test the hypotheses and could not be derived from reading each study separately. Demographics and comparisons between the two cohorts are summarized in Table 1. In the first cohort, 40 adult female white New Zealand rabbits had a surgical detachment of the SSP tendon followed by immediate reattachment of the SSP tendon with transosseous sutures. In the second cohort, 112 adult female white New Zealand rabbits had a surgical detachment of the SSP followed 1 week later by a reattachment with one anchor. Rabbits from the transosseous cohort were euthanized in groups of 10 immediately after surgery (0 week) or after 1, 2, or 6 weeks. Rabbits from the anchor cohort were euthanized in groups of 32 after 1, 2, or 4 weeks (after reattachment), and one group of 16 (32 shoulders) was euthanized immediately after surgery (0 week) (Table 1).

**Table 1:** Experimental cohorts

	Transosseous repair [21]	Anchor repair [22]
Repaired shoulders mechanically tested	*n* = 39	*n* = 105
Contralateral shoulders mechanically tested	*n* = 39	*n* = 105
Specie	New Zealand White rabbits	New Zealand White rabbits
Gender	100% female	100% female
Timing of repair	At tendon tear	1 week after tendon tear
Repair surgery	Open	Open
Anesthesia	Intramuscular ketamine, midazolam and glycopyrrolate Isofluorane anesthesia	Intramuscular ketamine, midazolam and glycopyrrolate Isofluorane anesthesia
Side	Alternating left and right shoulders	Alternating left and right shoulders
Additional interventions	None	- Microfracturing at SSP footprint - 50% bone channeling 1 week before repair - Distal tendon wrapped in polyvinylidene membrane for 1 week before repair
Suture	3–0 Prolene modifed Mason-Allen	#2 FiberWire horizontal mattress
Post-op analgesia	Fentanyl / buprenorphine × 3 days	Fentanyl / buprenorphine × 3 days
Post-operative duration	0, 1, 2 and 6 weeks	0, 1, 2 and 4 weeks
Average weight at harvest	3.51 ± 0.43 kg	3.03 ± 0.32 kg*
Mechanical testing	Cryogenic fixation/Cycling/Tensile testing to failure	Cryogenic fixation/Cycling/Tensile testing to failure

^*^p < 0.001 compared to transosseous repair cohort

### Surgical repair of the rotator cuff using transosseous suture

Under halothane anesthesia, a lateral skin incision followed by omovertebral and deltoid muscles’ retraction exposed the SSP tendon at its insertion into the greater tuberosity. The tendon was transected close to its insertion [16]. Reattachment surgery was performed immediately using the transosseous suture method. First, a 2 × 2 × 5 mm bony trough was made between the articular cartilage rim and the medial wall of the greater tuberosity using a burr. Three 1-mm tunnels were then drilled from the lateral aspect of the greater tuberosity into the bony trough. Two nonabsorbable 3-0 Prolene sutures were then placed as follows: the first thread was passed first through the most proximal drill hole, then through the distal tendon in a modified Mason-Allen fashion, and finally through the middle drill hole. The second thread was passed first through the middle drill hole, then through the distal tendon in a modified Mason-Allen fashion, and then through the distal drill hole. Both sutures were tied over the lateral aspect of the cortex, thus pulling the tendon stump into the trough. The wound was then closed in layers. Thus, the tendon stump was inserted into the footprint, the original site of the SSP insertion.

### Surgical repair of the rotator cuff using anchor suture

The surgical approach and SSP transection were identical to the transosseous repair cohort with the following additions (Table 1): The distal tendon was wrapped in a polyvinylidene membrane (5 μm, Durapore; Millipore, Bedford, MA) to prevent spontaneous postoperative reattachment. Bone channeling done in half of the rabbits involved dividing the SSP insertion footprint into four quadrants and a 1-mm diameter hole was drilled at the center of each quadrant to a depth of ~ 10 mm to communicate with the bone marrow. No channeling involved the same exposure, but no holes drilled. The repair surgery was performed 1 week later and was identical for channeled and not channeled shoulders. The incision was reopened. A curette was used to microfracture the footprint at the reattachment site. The retracted free distal SSP tendon stump was mobilized and the polyvinylidene membrane removed. A single 3-mm anchor (Bio-FASTak®; Arthrex, Naples, FL) with a braided polyethylene suture (#2 FiberWire) was inserted lateral and distal to the footprint in cortical bone [17]. The tendon was pulled over the lateral side of the greater tuberosity of the humeral head using a horizontal mattress stitch [23]. Thus, the articular side of the SSP contacted the outer humerus cortex but not the footprint. The end of the stump did not contact bone.

The rabbits were sourced commercially from Charles River (Saint Constant, Canada). Rabbits from both cohorts received fentanyl and buprenorphine for 3 days postoperatively and had unlimited access to food and water.

### Specimen collection

All animals were euthanized with a pentobarbital overdose. Both shoulders were dissected en bloc. The specimens were frozen at − 30 °C and underwent one freeze-thaw cycle for imaging before mechanical testing.

### Outcome assessment: biomechanical testing

The specimens were thawed gradually to room temperature. A cryogenic fixation unit (CFU) [21] secured the myotendinous junction of the tendon in saline ice using liquid nitrogen, while the proximal humerus was potted in bismuth alloy [22]. The specimen was then mounted on an electromechanical material testing system (MTS Sintech-1G; MTS Systems Corporation, Eden Prairie, MN). A circular heater was placed close to the CFU to keep the tendon at room temperature, monitored by one thermocouple integrated 5 mm below the CFU [17, 21, 23]. Petroleum jelly applied to the exposed tendon prevented dehydration. The tendons were tested in tension along their anatomic direction of pull, at an angle of 45° to the longitudinal axis of the humerus. Ten preconditioning cycles were conducted from an initial preload of 5 N to a peak load of 50 N at a loading rate of 15 N/s. After preconditioning, tensile loading to failure proceeded at a constant crosshead speed of 1 mm/s until a 50% drop in tensile strength stopped the test automatically. We distinguished between three modes of failure: suture pullout, bony avulsion, and midsubstance tendon tear. The load to failure was determined using TestWorks 4 software (MTS Systems Corporation). Stiffness was calculated by fitting a linear regression line between the toe region and peak load of the load-displacement curve for individual subjects.

### Data reduction and statistical analysis

Prehoc analysis (Table 1) revealed a significant difference of 0.48 kg in rabbit harvest weights between the two cohorts. To account for the potential effect of weight on the biomechanical properties of SSP tendons, we reported data from both cohorts as a percent of the contralateral unoperated side of the same rabbit. Anchor repairs showed no effect of channeling on load to failure, stiffness, or mode of failure thus all anchor repairs were analyzed as a single group [17]. In order to compare transosseous and anchor repairs at postoperative week 4, values were estimated by linearly interpolating rank-ordered pairs of transosseous repair load to failure and stiffness data between weeks 2 and 6.

A two-way ANOVA was applied to contralateral-normalized load to failure and stiffness data as dependent variables and repair type (transosseous repair vs anchor repair) and postoperative time (0, 1, 2, 4 weeks) as fixed factors. For the ANOVA, 1 datum missing in the transosseous repair cohort (control 2 weeks) was replaced with the sample average. Pairwise comparisons at each time point were performed using unpaired *t* tests. Mode of failure frequencies between transosseous and anchor repairs were compared using Chi-Square statistics. A *p* value ≤ 0.05 was considered statistically significant.

## Results

In the transosseous suture cohort, 2/80 shoulders (one operated and one control) and, in the anchor repair group, 14/210 shoulders (seven operated and seven control) were excluded from analysis due to tendon damage during surgery (4), during dissection (5), or mechanical testing (7). Final sample sizes are shown in Table 1.

### Load to failure

**Fig. 1:**
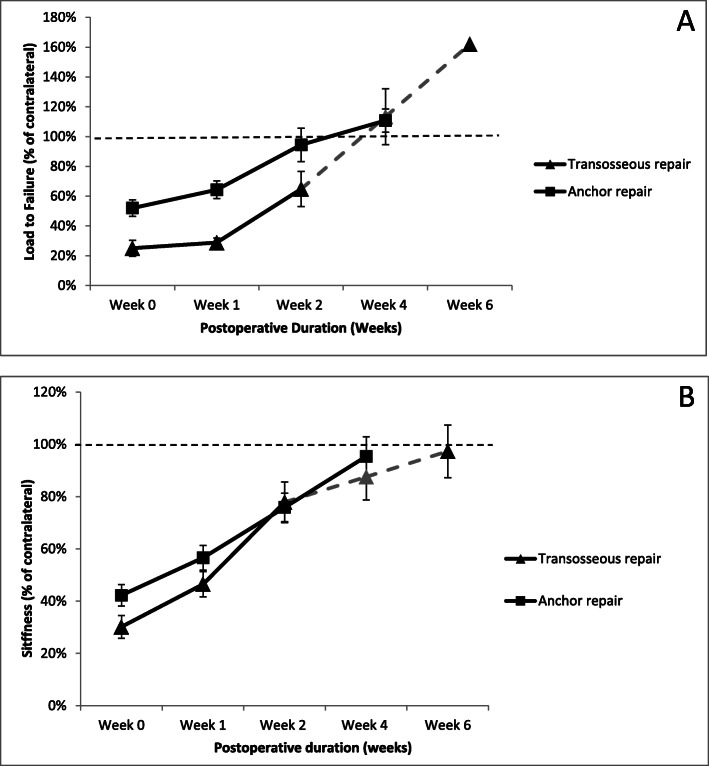
Normalized peak load at failure and stiffness of rabbit supraspinatus tendons up to 6 weeks after surgical transosseous or anchor repair. **a** Transosseous repair showed a steeper strength recovery curve between postoperative week 1 and 4 compared to anchor repairs indicating faster enthesis reformation. Anchor repairs were stronger at the time of surgery (week 0) and 1 week after repair. Both surgical techniques were comparable afterwards. **b** Stiffness was comparable between the 2 surgical methods at all postoperative durations. Both groups surpassed control load at failure and reached control stiffness. Data are expressed as percent of contralateral shoulder. Four-week data after transosseous repair (gray-discontinuous line) was interpolated from 2 and 6 week data (see the “Methods” section). *p = 0.004 compared to transosseous repair; ^#^*p* < 0.001 compared to transosseous repair. Error bars = 1 standard error of the mean

Load to failure was significantly associated with the repair technique (transosseous or anchor) (Fisher-Snedecor distribution (*F*) (*F* = 6.50; *p* = 0.011)) and postoperative time (week 0, 1, 2, or 4) (*F* = 21.73; *p* < 0.001) with no interaction between repair technique and postoperative time (*F* = 0.80; *p* = 0.497). Anchor repairs were significantly stronger than transosseous repairs both at initial surgery (52 ± 21% vs 25 ± 17%, respectively; *p* = 0.004) and at 1 postoperative week (61 ± 32% vs. 28 ± 10; *p* = 0.003; Fig. 1a). After 2 and 4 postoperative weeks, there were no statistically significant differences in load to failure between the two surgical repair techniques (*p* > 0.05; Fig. 1a). Load to failure increased with postoperative time in both techniques: transosseous (from 25 ± 17% at surgery to 113 ± 44% at week 4) and anchor (from 52 ± 21% at surgery to 113 ± 40% at week 4).

### Stiffness

Stiffness was not significantly associated with the repair technique (*F* = 1.95; *p* = 0.165) but was significantly associated with postoperative time (*F* = 21.57; *p* < 0.001) with no interaction between repair technique and postoperative time (*F* = 0.39; *p* = 0.759). Stiffness increased with postoperative time in both techniques: transosseous (from 30 ± 13% at surgery to 87 ± 23% at week 4) and anchor (from 42 ± 15% at surgery to 98 ± 35% at week 4; Fig. 1b).

### Mode of failure

**Fig. 2:**
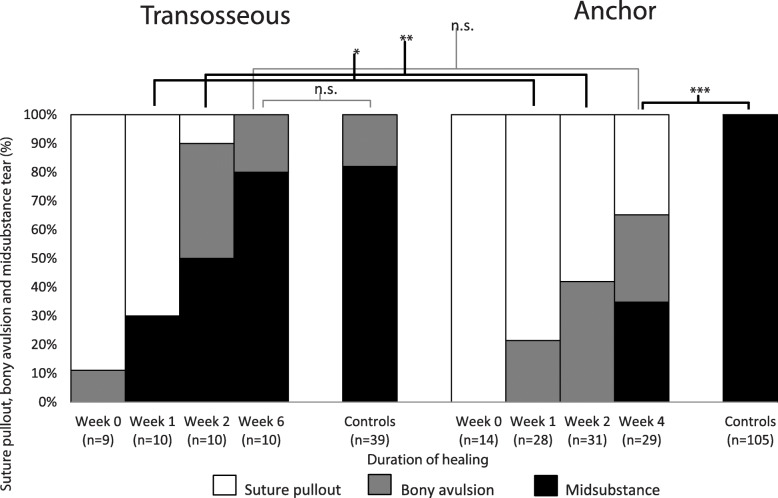
Mode of failure of rabbit supraspinatus tendons up to 6 weeks after surgical transosseous or anchor repair. Data are expressed as percentage (%) of the total sample at each postoperative duration (0, 1, 2, 4, or 6 weeks) for experimental and contralateral shoulders. Transosseous repairs showed a significantly higher proportion of midsubstance tendon tears at 1 (*p = 0.004) and 2 (**p < 0.001) postoperative weeks. Transosseous repairs showed at 2 postoperative weeks a similar proportion of midsubstance tears than anchor repair did after 4 postoperative weeks. The mode of failure in contralateral shoulders was midsubstance tendon tear. Transosseous repairs mode of failure did not significantly differ from controls after 4 postoperative weeks, while anchor repairs significantly did (***p < 0.001). n.s., non-significant difference

**Fig. 3:**
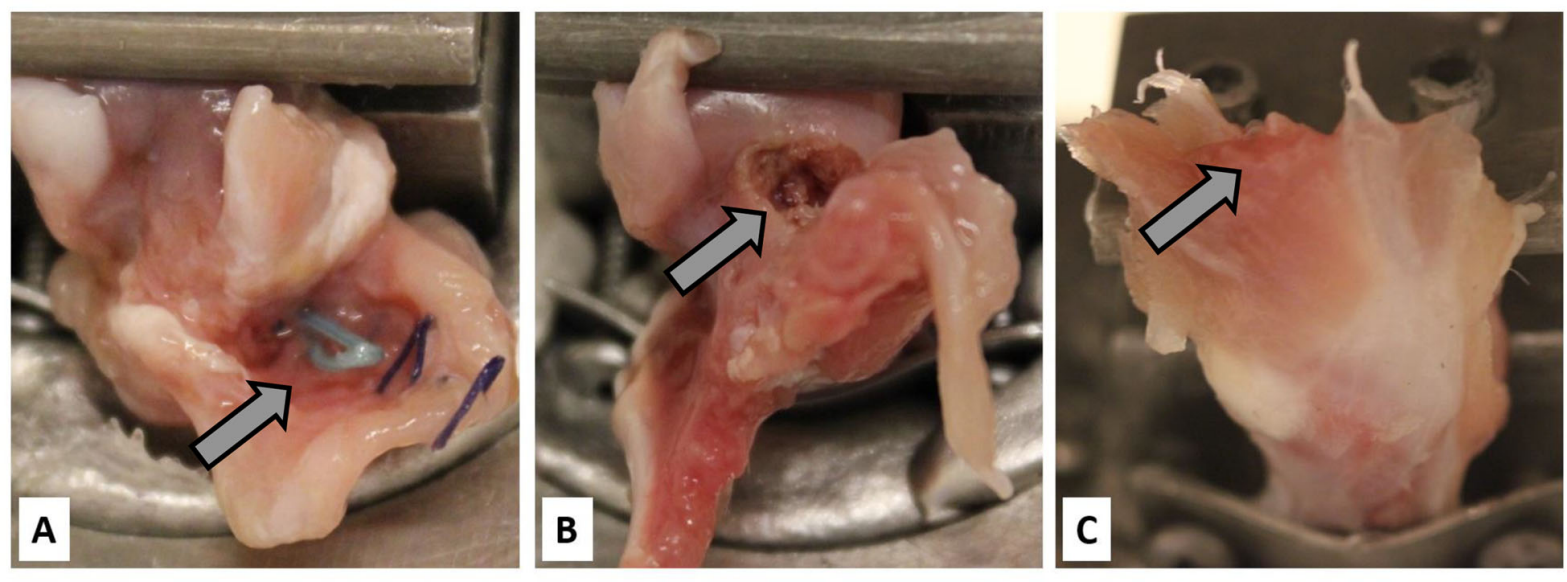
Mode of failure of rabbit supraspinatus tendons upon mechanical testing to failure. This figure shows the testing sites immediately after testing to failure illustrating the 3 modes of failure. **a** Suture pullout: arrow indicates the intact anchor suture. **b** Bony avulsion: arrow indicates area of bone defect. **c** Midsubstance SSP tendon tear: arrows indicate ruptured SSP tendon fibers

The mode of failure of control tendons in both cohorts was predominantly a midsubstance tear (Figs. 2 and 3). The mode of failure was significantly different between transosseous and anchor repairs at postoperative week 1 (*X*^2^ = 11.15; *p* = 0.004) and postoperative week 2 (*X*^2^ = 19.27; *p* < 0.001; Fig. 2). Transosseous repairs led to more midsubstance tendon tears than anchor repairs after 1 and 2 postoperative weeks (30% vs 0% and 50% vs 0%, respectively; Fig. 2). Transosseous repairs reached the same proportion of midsubstance tears as controls 6 weeks postoperatively (80% vs. 92%, respectively; *X*^2^ = 0.22; *p* = 0.881; Fig. 2). Anchor repairs did not reach the same proportion of midsubstance tears as controls 4 weeks postoperatively (32% vs 100%, respectively; *X*^2^ = 84.12; *p* < 0.001; Fig. 2). Finally, transosseous repairs at postoperative week 2 showed a comparable proportion of midsubstance tears as anchor repairs at postoperative week 4 (50% vs 32%, respectively; *X*^2^ = 2.08; *p* = 0.352; Fig. 2).

## Discussion

This study directly compared the biomechanical properties of two surgical techniques in 152 rabbit SSP tendons repairs and 152 controls. The difference between the two techniques was the site of contact of the SSP with bone: in transosseous repairs, the tendon stump was inserted into the footprint, its anatomical site, whereas in anchor repairs the articular side of the SSP contacted the lateral side of the greater tuberosity; the tendon stump did not participate in the healing process lying further distally to the site of repair. Anchor-repaired SSP were stronger than transosseous repairs at the time of surgery and at 1 postoperative week, confirming the first hypothesis. Both techniques reached comparable and full restoration of load at failure at postoperative week 4, refuting the second hypothesis that SSP anchor repairs reach normal loads at failure faster than transosseous repairs. Transosseous suture repairs, in turn, restored a midsubstance mode of failure characteristic of control tendons faster than anchor repairs. These data constitute the best powered comparisons of transosseous vs anchor mechanical properties of SSP tendons postoperatively.

We found only 2 related studies that compared postoperative SSP repairs in rabbits: in 2007, Wang et al. [24] found no difference in maximal loads at failure between transosseous vs anchor repairs with simple vs mattress sutures tested in 45 rabbits 8 weeks after repair, while in 2008, Ozbaydar et al. [25] reported higher loads to failure of double row compared to single row anchors in 80 rabbits at 4 and 8 postoperative weeks. However, all specimens failed at the site of repair at 5–9 N, 20-fold lower than results found in present samples [16, 17].

SSP repair seeks to relieve the patient symptoms and restore function. While symptom relief can be achieved with debridement only [26, 27], functional restoration is best achieved by re-establishing a lasting anatomical continuity between SSP tendon and humerus. At initial repair, the re-approximated distal tendon must contact the humerus footprint long enough for enthesis reformation to be initiated. However, the initial construct strength is significantly below the requirements for functional use of the arm (Fig. 1). A physical gap at the time of repair may never be bridged which would invariably lead to dehiscence. Postoperative gains in tensile strength are achieved by the progressive reformation of an enthesis between the distal SSP tendon and humerus. A reformed enthesis will allow a stronger SSP tendon. In turn, a strong SSP tendon will prevent a re-tear by suture pullout or bony avulsion when returning to activities. Re-tear, like dehiscence, would result in anatomical discontinuity [7].

In the current study, the stronger initial anchor repair may have benefitted from thicker, braided sutures allowing for greater force transmission compared to the thinner monofilament sutures of transosseous repair. As well, it may have benefitted from a mattress stitch [17] compared to the Mason-Allen stitch [16] of transosseous repair. However, we observed no tendon dehiscence with either technique. Thus, the difference in suture material had no influence on the outcome as no suture pullout nor suture rupture has been observed. The possibility of degeneration of the tendon stump occurring during the 1-week interval between tendon detachment and repair could not have affected the repair process as the stump was not the site of repair. This is consistent with the literature showing that delayed repair of 1–3 months neither adversely influenced the recovery of biomechanical properties nor enthesis reformation in the rabbit model [28].

At postoperative week 2, both transosseous and anchor-repaired shoulders exhibited comparable loads at failure. This implied accelerated enthesis reformation of the transosseous repair, effectively catching up to the stronger initial anchor repair (Fig. 1). The accelerated enthesis reformation between postoperative week 1 and 2 after transosseous repair may have benefitted from direct and prolonged contact between the distal SSP tendon and the exposed bone marrow and its reservoir of pluripotential mesenchymal stem cells (MSC) [29, 30]. Bone marrow-derived MSC may assist the cellular and extracellular matrix steps in reforming a new 4-zone enthesis. These steps include sequentially degrading the distal SSP tendon, differentiating and populating the distal SSP tendon with non-articular chondrocytes, reorganizing their alignment in rows and production of mineralized and non-mineralized fibrocartilage matrices [23, 31]. The anchor repair does not benefit from as large, direct, and prolonged a bone marrow exposure as the transosseous repair. Additional interventions of footprint microfracture and channeling in half of the shoulders in anchor repair cohort did not confer any significant benefit [17]. As the bone channeling in the anchor repair group was performed at the footprint, which did not participate in the healing process, it is of no wonder that an anticipated effect of marrow cells participating in the healing process could not be observed.

The mode of failure data added important insights into the initial construct and enthesis reformation in both repair techniques as it reflected the tensile resistance of various specialized tissue and material structures tested in series. Initially, suture pullouts must be entirely attributed to the surgical construct with no contribution from biological healing (Fig. 3). At postoperative weeks 1 and 2, enthesis reformation supplemented the surgical construct and the proportion of suture pullouts decreased at the benefit of midsubstance tears. This shift in mode of failure paralleled the restoration of the mechanical properties. The significantly higher proportion of midsubstance tears in transosseous repairs suggested they restored a physiological mode to failure significantly faster than anchor repairs. These data are compatible with the increased direct and prolonged contact between the distal SSP tendon and the exposed bone marrow.

After 4–6 postoperative weeks, the mechanical properties with both surgical techniques were restored. Interestingly, loads at failure above 100% of controls were recorded with both techniques at postoperative 4 and 6 weeks. Supranormal load at failure of repaired tendons cannot be attributed to weaker contralateral tendons since contralateral shoulders too increased their mechanical strength in both cohorts, possibly due to a training effect to compensate for the operated shoulder [16, 17]. Quite the opposite, the mechanical properties of operated tendons were compared to stronger contralateral. Reasons for the supranormal loads may include a postoperative footprint larger than the native footprint but we found no direct measure of postoperative SSP footprint size in the literature.

### Clinical implications

The surgical repair of supraspinatus tendon ruptures has moved from open, transosseous to arthroscopic, anchor based. A recent survey revealed that postoperative care in rotator cuff repair did not evolve in line with contemporary research evidence [32]. Evidence-based shoulder surgery can benefit from experimental data on SSP biomechanical restoration. Postoperative guidelines should consider the higher initial strength of anchor sutures and the faster recovery of strength of transosseous repairs rotator cuff repair techniques. In the debate between early versus delayed mobilization after rotator cuff repair, our findings of immediate tensile strength of anchor sutures double that of transosseous sutures would support a more dynamized “protected PROM” rehabilitation phase [33]. Then, the faster enthesis formation after transosseous repair would support an accelerated active mobilization phase after the protected phase.

Surgeons have supplemented anchor repairs with microfracturing, bone channeling of the SSP footprint or using hollowed anchors to achieve prolonged direct contact of the distal SSP tendon with exposed bone marrow [3, 34]. In the current study, despite all anchor-repaired SSP tendons receiving footprint microfracture and half receiving bone channeling at the footprint 1 week ahead of repair, none accelerated the biomechanical recovery in the rehabilitation phase compared to transosseous repair. These results found no evidence to suggest that these augmentation methods would affect surgical outcomes.

### Study limitations

Limitations inherent to animal models limit the generalization of the current findings. Contrary to humans, rabbit shoulders are weight-bearing. In both cohorts, a healthy SSP tendon was repaired, in contrast to degenerated tendons clinically. This study used an open repair and may cautiously be applied to a clinical setting with a preponderance of arthroscopic repairs. Follow-up was 4–6 weeks and allowed for full restoration of load at failure in both cohorts, decreasing the usefulness of studying later time points. Despite stated differences in protocols (suture material, surgical delay, channeling, unequal sample sizes, and postoperative time points), the data strongly supported the validity of the comparisons and their results. Other material properties, cross-sectional area, or thickness were not available to compare.

## Conclusion

We produce basic evidence that while anchor repairs provided superior initial strength of the SSP surgical construct, transosseous repair showed a faster speed of recovery of both strength and midsubstance mode of failure. Research into the ideal surgical repair could draw from the advantages of both techniques. Future work could use these data to refine repair strength simulation models allowing for the study and development of better designed surgical constructs.

## Data Availability

The datasets used and/or analyzed during the current study are available from the corresponding author on reasonable request.

## Abbreviations

SSP: Supraspinatus
CFU: Cryogenic fixation unit
MSC: Mesenchymal stem cells
